# Relevance of Biotin Deficiency in Patients with Inflammatory Bowel Disease and Utility of Serum 3 Hydroxyisovaleryl Carnitine as a Practical Everyday Marker

**DOI:** 10.3390/jcm11041118

**Published:** 2022-02-20

**Authors:** Johanna Erbach, Florian Bonn, Max Diesner, Anne Arnold, Jürgen Stein, Oliver Schröder, Ayşegül Aksan

**Affiliations:** 1 Interdisciplinary Crohn Colitis Center Rhein-Main, 60594 Frankfurt am Main, Germany; johannaerbach@gmail.com (J.E.); oschroeder@khs-ffm.de (O.S.); ayseguel.aksan@ernaehrung.uni-giessen.de (A.A.); 2 Immundiagnostik AG, 64625 Bensheim, Germany; florian.bonn@immundiagnostik.com (F.B.); max.diesner@immundiagnostik.com (M.D.); anne.arnold@immundiagnostik.com (A.A.); 3 DGD Kliniken Sachsenhausen, 60594 Frankfurt am Main, Germany; 4 Institute of Pharmaceutical Chemistry, Goethe University, 60438 Frankfurt am Main, Germany; 5 Institute of Nutritional Science, Justus-Liebig University, 35392 Giessen, Germany

**Keywords:** inflammatory bowel disease, biotin deficiency, 3-hydroxyisovaleryl carnitine

## Abstract

Background: Biotin, a water-soluble B vitamin, has demonstrable anti-inflammatory properties. A biotin-deficient diet induced a colitis-like phenotype in mice, alleviable by biotin substitution. Mice with dextran sulfate sodium (DSS)-induced colitis showed biotin deficiency and diminished levels of sodium-dependent multivitamin transporter, a protein involved in biotin absorption. Biotin substitution induced remission by reducing activation of NF-κB, a transcription factor involved in intestinal permeability and inflammatory bowel disease (IBD). We investigated for the first time a possible clinical role of biotin status in IBD. Methods: In a comparative, retrospective, cross-sectional study, serum samples of 138 patients with IBD (67 female; 72 Crohn’s disease (CD), 66 ulcerative colitis (UC)) aged 18–65 years and with a mean age (±SD) of 42.5 ± 14.3 years as well as 80 healthy blood donors (40 female; 40.0 ± 10.0 years; range 20–60 years) were analyzed. Inflammation was defined as hsCRP ≥5 mg/L, and to determine biotin status, serum 3-hydroxyisovaleryl carnitine (3HIVc) levels were measured by LC-MS/MS. Results: A total of 138 patients with IBD (67f; 72CD/66 UC; 42.5 ± 14.3 years) were enrolled: 83/138 had inflammation. Mean serum 3HIVc levels were significantly higher in IBD patients but unaffected by inflammation. Biotin deficiency (95th percentile of controls: >30 nmol/L 3HIVc) was significantly more common in IBD patients versus controls. Conclusion: High serum 3HIVc levels and biotin deficiency were associated with IBD but not inflammatory activity or disease type. Our findings suggest biotin may play a role as cause or effect in IBD pathogenesis. Routine assessment and supplementation of biotin may ameliorate IBD and support intestinal integrity.

## 1. Introduction

Biotin, a water-soluble B-complex vitamin, is an essential micronutrient [1,2] mainly known as a coenzyme for five human carboxylases (acetyl–CoA carboxylase (ACC) 1 and 2, pyruvate carboxylase (PC), propionyl–CoA carboxylase (PCC), and 3-methylcrotonyl–CoA carboxylase (MCC)) [2,3]. Its traditional role is as a covalently bound coenzyme for carboxylases utilized in various cellular metabolic pathways, such as fatty acid synthesis and oxidation, gluconeogenesis, the degradation of branched-chain amino acids and odd-chain fatty acids, and leucine catabolism [2,4,5]. Lately, however, it has also been recognized for its noncarboxylic biological functions, including cell signaling [6], epigenetic regulation of genes [7,8,9] and chromatin structure [8,9], and more recently, immune response [3,10,11,12,13].

Studies on biotin’s role in the immune response have emergently revealed that biotin is related to inflammation [10,14,15] and that its deficiency leads to an increase in the level of proinflammatory cytokines [13,16]. Skupsky et al. reported that diet-induced biotin deficiency in mice leads to increased concentrations of the fecal calprotectin, an inflammatory marker used routinely to monitor the progression of patients with inflammatory bowel disease (IBD). Importantly, they also showed that these mice fully recover from their IBD-like state under biotin supplementation [17].

IBD is characterized by chronic relapsing and remitting inflammation of the gastrointestinal tract [18,19]. Although the exact pathogenesis of IBD remains unclear, it has shown to be associated with genetic predisposition in combination with a complex variety of environmental influences (e.g., diet, smoking, sleep) in addition to microbial and immune factors [20,21,22]. Since IBD is as yet incurable, efficient remission maintenance is the major goal of therapy. Therefore, it is crucial to identify triggers and inhibitors of inflammation. Many studies have demonstrated possible effects of various vitamins and minerals as causes and/or consequences of inflammation in IBD [23,24,25,26,27,28,29,30,31,32,33,34,35]. Specific individual micronutrients have been the subject of huge interest; these include vitamin D due to its immunological effects [36,37,38] and iron, folate, and vitamin B12 due to their importance in anemia, the most common extraintestinal manifestation of IBD [39,40,41]. In contrast, other micronutrients, such as biotin, have remained in the shadows and have yet to be comprehensively researched. Although current evidence on biotin in relation to IBD originates largely from preclinical studies, the findings from these studies are solid and highly promising [10,13,14,15,16,17]. However, since there are very few clinical studies, it is unclear whether these preclinical findings are an accurate reflection of real-life experience in the clinic. In this study, we therefore aimed to take a very first step by ascertaining a cut-off to define biotin deficiency on the basis of serum 3-hydroxyisovaleryl carnitine (3HIVc) concentrations [42], to assess 3HIVc levels in patients with IBD, and to compare these levels in patients with versus without clinical evidence of inflammatory activity and with healthy controls.

## 2. Patients and Methods

This study was conducted as a comparative, retrospective, cross-sectional study in adult patients previously diagnosed with IBD and healthy blood donors, according to the guidelines laid down in the Declaration of Helsinki. The study design was approved by the local ethics committee of the Landesärztekammer Hessen (FF33/2019). All patients were required to give written informed consent as confirmation that they had been personally informed about all aspects of the research, including data protection measures.

### 2.1. Study Population

Serum samples from patients aged 18–65 years who consecutively attended the Interdisciplinary Crohn Colitis Center Rhein-Main, Frankfurt am Main, Germany, between August 2020 and August 2021 and who had been diagnosed with IBD at least 6 months previously according to standard clinical, radiological, and pathological criteria [43,44] were analyzed for parameters of inflammation and additionally for 3HIVc as part of diagnostic blood tests. Exclusion criteria for the study included pregnancy, lactation, recent history of smoking, inborn errors of biotin metabolism, use of vitamin supplements, and use of anticonvulsants. All patients who were potentially eligible on the basis of the stated criteria were personally informed about study procedures and data protection measures and gave written informed consent prior to inclusion of their data in the analysis. The control group consisted of 80 serum samples from healthy blood donors, acquired from the local blood bank.

### 2.2. Study Design

Demographic data, disease characteristics, and details of ongoing treatment were obtained and documented from patient medical records. Blood samples were collected between 08:00 and 10:00 h during routine follow-up visits, and serum samples were stored at −30 °C until analysis.

Blood count, serum high-sensitivity C-reactive protein (hsCRP) levels, and fecal calprotectin concentrations were analyzed according to the guidelines of the German United Society for Clinical Chemistry and Laboratory Medicine (Deutsche Vereinte Gesellschaft für Klinische Chemie und Laboratoriumsmedizin, DGKL) at a local reference laboratory (Laborarztpraxis Dres. med. Walther, Weindel und Kollegen MVZ GbR, Frankfurt am Main, Germany). Serum 3HIVc concentrations were measured by liquid chromatography–mass spectrometry/mass spectrometry (LC-MS/MS) using the IDK^®^ KMR8100 LC-MS/MS kit (Immundiagnostik, Bensheim, Germany) according to the manufacturer’s instructions. Results were expressed in nmol/L.

The patients’ samples were assessed for presence or absence of inflammatory disease activity on the basis of detected levels of hsCRP (normal value defined as <5 mg/L) and fecal calprotectin (normal value defined for patients with IBD as 250 µg/g) [45,46]. High values of either hsCRP or fecal calprotectin or both were taken to be signs of inflammatory activity.

### 2.3. Defining Functional Biotin Deficiency

More than 20 different markers of biotin status have been described. These are typically classified as either direct or indirect markers. 3HIVc is an indirect marker that rises in response to diminished activity of the methylcrotonyl-coenzyme, a biotin-dependent enzyme [47]. Analysis of serum 3HIVc levels by LC-MS/MS has been found to be one of the most sensitive markers of biotin depletion [42]. However, to our knowledge, no clear clinical cut-off value has previously been defined for functional biotin deficiency. Therefore, we calculated the predicted reference range for serum 3HIVc levels by calculating percentile values within the control group and took the 95th percentile as a cut-off for functional biotin deficiency.

### 2.4. Statistical Analysis

The statistical data were collected in Microsoft Office 365 Excel and analyzed using IBM’s Statistical Package for the Social Sciences (SPSS) in the version SPSS Statistics 25. All the variables were investigated for normal distribution using both visual (histograms, probability plots) and analytical (Kolmogorov–Smirnov test) methods. The values are presented as mean ± standard deviation for normally distributed variables and otherwise as median (min–max). For comparison of the independent groups, appropriate parametric or non-parametric tests were performed based on a normal distribution using Student’s *t*-test or Mann–Whitney U tests. Chi-square tests were used to define the relationships between categorical variables. Statistical significance was predetermined as *p* < 0.05.

## 3. Results

### 3.1. Study Population

In total, 138 patients with IBD (67 female, 71 male; 72 Crohn’s disease (CD), 66 ulcerative colitis (UC)) who were aged 18–65 years with a mean age (±SD) of 42.5 ± 14.3 years and who attended the IBD center between August 2020 and August 2021 were included in the study. Samples from these patients were analyzed and compared with a control group comprising serum samples from 80 healthy blood donors (40 male, 40 female; 40.0 ± 10.0 years; range 20–60 years). In the patient group, 83/138 (60%) had active disease (hsCRP ≥ 5 mg/L and or fecal calprotectin ≥ 250 µg/g). Characteristics of the patients and controls are shown in Table 1.

### 3.2. 3HIVc Levels

Serum 3HIVc levels of patients and controls are presented in Table 2. Patients with IBD were found to have significantly higher 3HIVc levels than controls (median 20.1 (5.8–79.4) vs. 17.3 (7.4–42.6), *p* = 0.024).

3HIVc concentrations were compared according to different disease characteristics within the patient group: Serum 3HIVc levels were found to be similar in patients with CD and UC (20.1 (6.2–59.2) vs. 19.8 (5.8–79.5), *p* = 0.848). In patients with CD grouped according to disease localization according to the Montreal Classification, no statistically significant differences in 3HIVc levels were observed (18.5 (7.7–59.0) for L1; 20.3 (7.3–49.4) for L2; 19.3 (6.2–39.0) for L3; and 23.2 (12.0–59.2) for L4, *p* = 0.713). Similarly, in patients with UC, disease localization was not found to be related to serum 3HIVc levels (29.4 (15.9–41.9) for E1; 19.3 (7.3–49.4) for E2; and 19.4 (5.8–79.4) for E3, *p* = 0.298). Patients were also found to have similar serum 3HIVc levels irrespective of whether or not inflammatory activity was present (20.0 (6.2–79.4) vs. 20.6 (7.3–59.2), *p* = 0.904).

Similarly, when disease type and inflammatory status were taken together, no statistical differences were detected for serum 3HIVc levels: In patients with CD and inflammatory activity, the median serum 3HIVc concentration was 20.0 (6.2–55.8) compared with 20.6 (7.3–59.2) in patients with CD with no evidence of active inflammation (*p* = 0.904). Likewise, serum 3HIVc levels did not differ in patients with UC with versus without inflammation (19.8 (5.8–79.4) vs. 20.4 (7.2–50.6), *p* = 0.483).

### 3.3. 3HIVc Cut-Off Value

**Figure 1:**
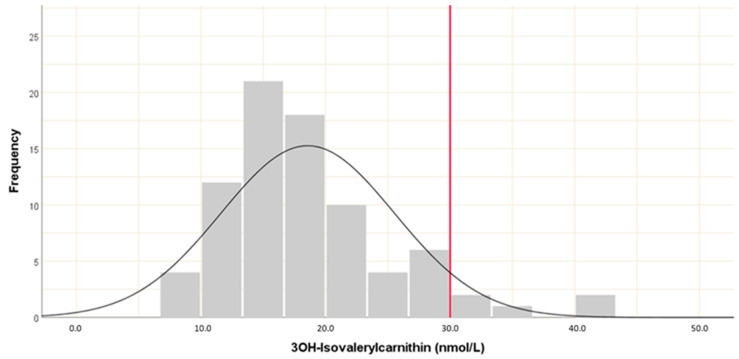
Predicted reference value for serum 3HIVAc levels from control samples.

A reference value for 3HIVc was calculated from the serum levels of healthy blood donors in the control group. The 95th percentile was found to equal a level of 30 nmol/L, which was therefore taken as the healthy cut-off point, with higher values taken to represent functional biotin deficiency (Figure 1).

### 3.4. Predicting Functional Biotin Deficiency on the Basis of Serum 3HIVc Concentrations

The prevalence of biotin deficiency in study subjects with IBD in comparison to the controls is presented in Table 3. Biotin deficiency was detected in 21.7% of patients in the IBD group and thus in a significantly higher proportion compared with the control group (*p* = 0.007).

On the other hand, the frequency of biotin deficiency was similar between patients with CD (20.8%) and UC (22.7%), between different disease localizations (29.2% for L1, 25.0% for L2, 13% for L3, and 25% for L4 in CD; *p* = 0.601, 50% for E1, 18.4% for E2, and 15% for E3 in UC; *p* = 0.263), between patients with (24.1%) vs. without (18.2%) inflammatory activity (*p* = 0.410), and between subclasses combining disease type and inflammation status (with inflammation 25.6%; without inflammation 13.8% for CD, *p* = 0.227; with inflammation 22.5%; without inflammation 23.1% for UC, *p* = 0.956).

## 4. Discussion

Despite the prevailing assumption that biotin deficiency is rare, there is mounting evidence indicating the existence of several physiological and pharmacological states in which biotin status is compromised. Other than primary causes, such as neonatal biotinidase deficiency or inborn errors of biotin metabolism, biotin deficiency has been associated with such diverse factors as protein-energy malnutrition, long-term parenteral nutrition, anticonvulsant therapy, pregnancy, alcoholism, smoking, and aging [48,49,50,51,52,53,54,55,56,57].

Although conclusive evidence is lacking, some data indicate that biotin deficiency might also occur more frequently in the context of IBD than in the healthy population [58,59]. However, the studies of Fernandez-Banares et al. [58] and Abad Lacruz et al. [59] on biotin deficiency in patients with IBD, both from the same study group, were published long before the role of biotin in inflammation and immunity came to light. In both studies, the sample size of IBD patients was small, at 23 and 8, respectively, and the research focused mainly on vitamin deficiencies rather than biotin deficiency. Abad Lacruz et al. [59] concluded that biotin deficiency might be related to protein energy malnutrition rather than IBD itself since at that time (1988), patients with IBD often had protein energy malnutrition at the time of hospital admission. Today, mounting evidence points to a deeper relation between biotin status and IBD, which is, however, yet to be understood.

Since mammals are unable to synthesize biotin endogenously, they absorb this essential micronutrient in two different regions of the gastrointestinal tract from two different exogenous sources: Firstly, dietary biotin (the major dietary sources including offal, eggs, fish, meat, seeds, nuts, and certain vegetables) is processed and subsequently absorbed in the small bowel. Secondly, biotin is produced by the gut microbiota in the large bowel, where it is additionally absorbed [60,61,62,63]. The sodium-dependent multivitamin transporter (SMVT) plays an important role in the Na-dependent, carrier-mediated process of biotin absorption in both the small and the large bowel [64,65]. Molecular identity of the SMVT mechanism has been characterized using cloning techniques that showed this carrier protein to be expressed by the SLC5A6 gene [66,67].

In preclinical studies with slc5a6 gene-knockout mice, Ghosal et al. [68] showed that SMVT is the sole carrier responsible for biotin absorption. Interestingly, it was also reported that, in these mice, biotin deficiency led to histological abnormalities in the small bowel (shortened villi, dysplasia) and cecum (chronic active inflammation, dysplasia). The same study group went on to investigate the role of SMVT in inflammation and showed that diet-induced biotin deficiency promotes the development of IBD-like chronic active inflammation in the cecum, accompanied by increased expression of proinflammatory cytokines, increased permeability of the gut wall, and altered expression levels of the tight junction proteins, again, imitating an IBD-like state. They also highlighted the role of intestinal SMVT in maintaining normal mucosal integrity, presumably by distributing biotin to the various cells that constitute the intestinal mucosa [15]. In line with these findings, Kuroishi et al. [13] demonstrated in murine models that a deficiency or excess of biotin, respectively, may up- or downregulate expression of TNF-α, suggesting that biotin status may be an influential factor in inflammatory disease. More recently, Skupsky et al. [17] reported that oral biotin supplementation shows benefit in the induction and maintenance of remission in a dextran sodium sulphate rodent model of IBD by suppressing activation of NF-kB, thus hindering the expression of inflammatory cytokines and helping to maintain integrity of the endothelial barrier of the gut wall. Since the NF-kB pathway is known to be clinically important in the etiology of IBD, this finding suggests that a possible therapeutic potential of biotin in patients with IBD may be well worth investigating.

The sum of the preclinical findings highlighting associations of biotin with IBD through a role in both inflammation and mucosal integrity encouraged us to search for similar relations in the clinical setting. To our knowledge, ours was the first study specifically focused on biotin deficiency in patients with IBD. As an additional, secondary outcome, we defined a diagnostic cut-off for one of the common diagnostic markers for biotin depletion, serum 3HIVc, to define functional biotin deficiency. We decided to use serum as our sample medium due to the practicability of testing during routine blood tests. However, we found no clear, existing cut-off in the literature for normal serum values of 3HIVc as a marker of functional biotin deficiency in healthy adults. In 2010, Stratton et al. conducted a study investigating plasma 3HIVc levels in healthy adults who underwent 2 weeks of biotin supplementation (300µg/day) followed by a 28-day washout period (low-biotin diet with undenatured avidin in egg white) and a final repletion phase with renewed biotin supplementation, in which 3HIVc was found to be suitable as a marker to detect marginal biotin deficiency [47]. However, the study had a small sample size, with only 10 subjects (8 female). The authors took the full range of values (0.002 to 0.008 µmol/L) at day 0 (biotin repletion) as a reference range. In our study, the serum 3HIVc cut-off value, defined as the 95th percentile of levels detected in our control samples, was considerably higher than the plasma levels defined by Stratton and colleagues, at 30 nmol/L (0.03 µmol/L). This may be at least partially due to a lack of harmonization of the assays used but could also reflect the fact that our controls were not necessarily biotin replete. On the other hand, our control group was considerably larger (n = 80), had equal numbers of males vs. females, and was therefore presumably representative of a cross-section of adults without any prior intervention to replete or restore biotin to ideal levels.

In parallel with preclinical findings, we found patients with IBD to have significantly higher 3HIVc levels than controls. However, we found no differences according to disease type, disease localization, or more surprisingly, the presence of inflammatory activity. Similarly, when we classified subjects by biotin status according to the cut-off value of serum 3HIVc levels defined by the 95th percentile defined from our control samples (30 nmol/L), the prevalence of functional biotin deficiency was significantly higher in the IBD group in comparison to the control group but did not differ according to disease type, localization, or inflammation status.

A possible limitation of the study might be the size of the patient groups after classification according to disease localization. Additionally, it would be helpful and statistically sounder to have a larger group of control subjects from which to determine the clinical cut-off for serum 3HIVc levels. Screening opportunities were unfortunately limited as a result of reduced availability of study staff to inform and screen patients due to the SARS-CoV-2 pandemic, including effects of related national, regional, and institutional safety measures. The nutritional status of patients at the time of blood collection was not documented, neither were any nutritional preconditions set for the patients prior to study participation. This may be considered a weakness of the study. However, it is well known that since biotin is universally available and additionally produced by intestinal bacteria, biotin deficiency as a result of malnutrition is very rare; more commonly, it may occur in patients with short bowel syndrome or intestinal malabsorption, patients taking antibiotics, or those receiving long-term parenteral nutrition. Avidin, found in raw egg white, can bind biotin and thus occasionally cause deficiency in people who consume large amounts of raw egg in their diet [1,69]. The three autosomal recessive syndromes, referred to as “multiple carboxylase deficiency,” holocarboxylase synthetase deficiency (the “neonatal form”), biotinidase deficiency (the “juvenile form”), and the rare syndrome of inability to transport biotin into cells, all have similar clinical features and can cause biotin deficiency [1]. Our focus in this study was to discover whether the prevalence/severity of biotin deficiency is increased as a result of IBD-related gastrointestinal symptoms. It has been proposed that biotin deficiency may exacerbate the inflammatory response from dendritic cells, causing excessive inflammation. Therefore, if indeed biotin deficiency occurs as a result of IBD and at the same time triggers and/or intensifies inflammation itself, this could cause a perpetual vicious circle of worsening inflammation that needs to be treated. Nevertheless, in future, based on the promising results of this initial exploratory analysis, well-designed prospective studies are required that take into account the patients’ nutritional status and include additional inflammatory markers to better reflect inflammatory activity overall. On the other hand, this study is the first to investigate the association between biotin status and IBD in a clinical setting, focusing at the same time on possible interactions of biotin status with inflammation as well as disease type and localization.

In conclusion, the findings of this study are in line with the existing preclinical evidence indicating that biotin deficiency is more common in patients with IBD in comparison to healthy controls in a clinical setting. However, somewhat surprisingly, the biotin status of patients with IBD was not found to differ according to inflammatory status. Therefore, the next step in clinical biotin and IBD research could be to take a more comprehensive look into the relationship between IBD-related inflammation and biotin deficiency.

## Figures and Tables

**Table 1:** Characteristics of patients and controls.

	Patients with IBD		Control Group ^†^	p_1_	p_2_
	Crohn’s Disease	Ulcerative Colitis			
**N (female)**	72 (33)	66 (34)	80 (40)	0.505	0.837
**Age (mean ± sd)**	40.6 ± 14.5	44.6 ± 13.8	40.0 ± 10.0	0.096	-
**Age at IBD diagnosis ^#^, n (%)**					
A1 = Below 16 years	13 (18.1)	6 (9.1)	-	0.162	-
A2 = 17 to 40 years	42 (58.3)	42 (63.6)			
A3 = Over 40 years	9 (12.5)	14 (21.2)			
**Disease localization ^#^, n (%)**					
L1 = Terminal ileum (CD)	24 (33.3)	-	-	-	-
L2 = Colon (CD)	8 (11.1)				
L3 = Ileocolon (CD)	23 (31.9)				
L4 = Upper gastrointestinal tract (CD)	8 (11.1)				
E1 = Proctitis (UC)	-	4 (6.1)	-	-	-
E2 = Left-sided colitis (UC)		38 (57.6)			
E3 = Pancolitis (UC)		20 (30.3)			
**Disease behavior ^#^ (CD), n (%)**					
B1 = Nonstricturing, nonpenetrating	31 (43.1)	-	-	-	-
B2 = Stricturing	12 (16.7)				
B3 = Penetrating	19 (26.4)				
B4 = Perianal	1 (1.4)				
**Current medication, n (%)**					
No treatment	6 (8.3)	3 (4.5)	-	0.476	-
5-ASA	2 (2.8)	16 (24.2)		<0.001 **	
Corticosteroids	13 (18.1)	25 (37.9)		0.013 *	
Immunomodulators	28 (38.9)	29 (43.9)		0.605	
Biologicals	19 (26.4)	23 (34.8)		0.355	

* *p* < 0.05, ** *p* < 0.001 (for age, Student’s *t*-test; and for gender, age at diagnosis, and medication, chi-square test). CD, Crohn’s disease; UC, ulcerative colitis. ^†^ Control group consisting of healthy blood donors aged 20–60. ^#^ Age at IBD diagnosis, disease localization, and disease behavior (Crohn’s disease only) characterized according to Montreal Classification of IBD. p_1_: statistical significance between patients with Crohn’s disease and patients with ulcerative colitis; p_2_: statistical significance between patients with IBD in total and control group.

**Table 2:** Comparison of serum 3HIVAc levels according to disease type and inflammatory activity.

	N	3HIVAc (nmol/L) Median (Min–Max)	*p*
**Disease status**			
Patients with IBD	138	20.1 (5.8–79.4)	
Controls	80	17.3 (7.4–42.6)	
p_1_			0.024 *
**Disease type**			
Crohn’s disease	72	20.1 (6.2–59.2)	
Ulcerative colitis	66	19.8 (5.8–79.5)	
p_2_			0.848
**Disease location ^#^**			
*Crohn’s disease*			
L1 = Terminal ileum	24	18.5 (7.7–59.0)	
L2 = Colon	8	20.3 (7.3–49.4)	
L3 = Ileocolon	23	19.3 (6.2–39.0)	
L4 = Upper gastrointestinal tract	8	23.2 (12.0–59.2)	
p_3_			0.713
*Ulcerative colitis*			
E1 = Proctitis	4	29.4 (15.9–41.9)	
E2 = Left-sided colitis	38	19.3 (5.8–50.6)	
E3 = Pancolitis	20	19.4 (5.8–79.4)	
p_4_			0.298
**Inflammatory status**			
Inflammatory activity	83	20.0 (5.8–79.4)	
No inflammation	55	20.6 (7.2–59.2)	
p_5_			0.731
**Disease type and inflammatory status**			
*Crohn’s disease*			
Inflammatory activity	43	20.0 (6.2–55.8)	
No inflammation	29	20.6 (7.3–59.2)	
p_6_			0.904
*Ulcerative colitis*			
Inflammatory activity	40	19.8 (5.8–79.4)	
No inflammation	26	20.4 (7.2–50.6)	
p_7_			0.483

* *p* < 0.05 (Mann–Whitney U test) ^#^ Characterization of disease localization according to Montreal Classification of IBD. p_1_: statistical significance between patients with IBD in total and control group; p_2_: statistical significance between patients with Crohn’s disease and patients with ulcerative colitis; p_3_: statistical significance between L1, L2, L3, and L4 localization types in patients with Crohn’s disease; p_4_: statistical significance between E1, E2, and E3 localization types in patients with ulcerative colitis; p_5_: statistical significance between patients with IBD with vs. without inflammatory activity; p_6_: statistical significance between patients with Crohn’s disease with vs. without inflammatory activity; p_7_: statistical significance between patients with ulcerative colitis with vs. without inflammatory activity.

**Table 3:** Frequency of biotin deficiency in patients with IBD in comparison to controls.

	N	Serum 3HIVAc Levels	
		Optimal (<30.0 nmol/L) N (%)	High (≥30.0 nmol/L) N (%)
**Disease status**			
IBD patients	138	108 (78.3)	42 (21.7)
Control	80	76 (95.0)	4 (5.0)
p_1_		0.007 *	
**Disease Type**			
Crohn’s Disease	72	57 (79.2)	15 (20.8)
Ulcerative Colitis	66	51 (77.3)	15 (22.7)
p_2_		0.838	
**Disease Location**			
*Crohn’s Disease*			
L1 = Terminal ileum	24	17 (70.8)	7 (29.2)
L2 = Colon	8	6 (75.0)	2 (25.0)
L3 = Ileocolon	23	20 (87.0)	3 (13.0)
L4 = Upper gastrointestinal tract	8	6 (75.0)	2 (25.0)
p_3_		0.601	
*Ulcerative Colitis*			
E1 = Proctitis	4	2 (50.0)	2 (50.0)
E2 = Left-sided colitis	38	31 (81.6)	7 (18.4)
E3 = Pancolitis	20	17 (85.0)	3 (15.0)
p_4_		0.263	
**Inflammatory status**			
Inflammatory activity	83	63 (75.9)	20 (24.1)
No inflammation	55	45 (81.8)	10 (18.2)
p_5_		0.410	
**Disease type and inflammatory status**			
*Crohn’s disease*			
Inflammatory activity	43	32 (74.4)	11 (25.6)
No inflammation	29	25 (86.2)	4 (13.8)
p_6_		0.227	
*Ulcerative colitis*			
Inflammatory activity	40	31 (77.5)	9 (22.5)
No inflammation	26	20 (76.9)	6 (23.1)
p_7_		0.956	

* *p* < 0.05 (Chi-square test) p_1_: statistical significance between IBD patients in total and control, p_2_: statistical significance between patients with Crohn’s disease and patients with ulcerative colitis, p_3_: statistical significance between L1, L2, L3, and L4 localization types in patients with Crohn’s disease; p_4_: statistical significance between E1, E2, and E3 localization types in patients with ulcerative colitis; p_5_: statistical significance patients with IBD with vs. without inflammatory activity; p_6_: statistical significance between patients with Crohn’s disease with vs. without inflammatory activity; p_7_: statistical significance between patients with ulcerative colitis with vs. without inflammation.

## Data Availability

The data presented in this study are available on request from the corresponding author. The data are not publicly available due to national/international data protection requirements.
